# 

**Published:** 1899-06-17

**Authors:** 


**Notices of Births, Deaths, and Marriages** are inserted in this column at a charge of 2s. 6d. for an announcement not exceeding four lines.

### NOTICE TO CORRESPONDENTS.

All MS., Letters, Books for Review, and other matters intended for the Editor should be addressed The Editor, "The Hospital," The Hospital Building, 28 & 29, Southampton Street, Strand, W.C.
The Editor cannot undertake to return rejected MS., even when accompanied by stamped directed envelope.

**Notice to Advertisers and Subscribers.**

All Advertisements, Orders for Copies of The Hospital, and Business Communications should be addressed to **THE MANAGER (Not to the Editor)**, The Hospital, The Hospital Building, 28 & 29, Southampton Street, Strand, London, W.C.
The Cost of Subscription, post free, is as follows:—Per week, 2½d.; per quarter, 2s. 8d.; per half-year, 5s. 3d.; per annum, 10s. 6d. Foreign:—Per annum, 15s. 2d., payable, in all cases, in advance.

**NOTICE.—Vol. XXV. of "The Hospital" (October, 1898, to March, 1899), in handsome cloth binding, is now on sale at the Office of this Journal, price 7s. 6d. Cases for binding the Numbers contained in this Volume 1s. 6d. Index to Vol. XXV., 2½d., post free.**
**The Monthly Part for July will be ready on July 1st. Price 6d., by post 8d.**

### BOOKS RECEIVED.

Spottiswoode and Co.

"A Provident Dispensary and Nursing Association." By Jamieson B. Hurry, M.A., M.D.

E. Gould and Son.

"Viscum Album." By George Black, M.B.

Swan Sonnenschein and Co.

"The Elements of Vital Statistics." By Arthur Newsholme, M.D., F.R.C.P.

Sampson Low, Marston, and Co.

"Handbook of Medical Gymnastics." By Anders Wide, M.D.

Baillière, Tindall, and Cox.

"The Pocket Pharmacopœia." By Frederick Hudson-Cox., M.I.C., F.C.S., and John Stokes, M.D., B.S., L.S.Sc., M.R.C.S.

**Periodicals and Pamphlets.**—Guy's Hospital Gazette, Asylum News, Medical Record, St. Bartholomew's Hospital Journal, Leeds Hospital Magazine, Zenana, Medical Press, Columbus Medical Journal, Medical Temperance Review, Guy's Hospital Gazette, St. Thomas's Hospital Gazette, Public Health Journal, Metaphysical Magazine, London Hospital Gazette, English Illustrated Magazine, Cassell's Magazine, Medical News, Literary Digest, Local Government Journal, Municipal Journal, Medical and Surgical Review of Reviews, The Art Portfolio.

